# Responsiveness of the Sensor Network to Alarm Events Based on the Potts Model

**DOI:** 10.3390/s20236979

**Published:** 2020-12-06

**Authors:** Andrzej Paszkiewicz, Jan Węgrzyn

**Affiliations:** 1 Department of Complex Systems, The Faculty of Electrical and Computer Engineering, Rzeszow University of Technology, 35-959 Rzeszów, Poland; 2 The Faculty of Electrical and Computer Engineering, Rzeszow University of Technology, 35-959 Rzeszów, Poland; 148915@stud.prz.edu.pl

**Keywords:** Potts model, Ising model, sensor networks, complex systems, Industry 4.0

## Abstract

The paper aims to present modelling the sensor network operation based on the Potts model. The authors presented own approach based on three states in which each node can be. The change in the state of a given node depends on its current state, the impact of surrounding nodes on it, but also values of the parameters measured. Therefore, the Hamiltonian was introduced as a dependence of both exceeding the limit value of a measured parameter (corresponding to an alarm event), and the state of the battery powering a given node of a sensor. The simulations of the implemented algorithm based on the adopted model presented in the paper relate to the measurement of temperature by a network of sensors. However, this model is universal and can be applied to examine the behaviour of the sensor infrastructure performing various measurements. Moreover, it may simulate the functioning of the critical network infrastructure or sensor networks and industrial sensors supporting the functioning of Industry 4.0.

## 1. Introduction

Sensor networks are a very important element of the modern IoT infrastructure in the civil, military and industrial area [1,2,3,4,5,6,7,8,9,10]. They are able to collect and pass on a lot of important information. Some of this data can be processed by the terminal device itself, while the other are subject to more extensive analysis. A more complex implementation of the concept of Industry 4.0, the development of the Internet of Everything (IoE) and the Internet of Things (IoT) will not be possible without expansion and modernization of i.e., sensor network infrastructure. Obviously, it includes such elements as sensors, measurement and communication technologies, application and data analysis layer, etc. Sensor network systems should be considered in terms of complex systems theory due to their multifaceted nature [11,12,13]. Their characteristics includes such features as self-organization, self-adaptation, as well as non-linearity, nonequilibrium thermodynamics, non-extensiveness, non-stationarity of processes, sensitivity to overloads and infections, emergence, dynamic load balancing, scale-free processes, fractional Brownian motion, power laws, small worlds, preferential attachment, non-extensive statistics etc. [14,15,16]. Incorporating all above mentioned features seems a very complex task as of today. Therefore, complex systems are analyzed in the context of single or limited number of features at this stage of the work. The phase transition phenomenon is one of complex system properties associated with a rapid change in the values of important parameters of a given object usually leading to a change of its properties. This type of phenomena is analyzed with the Potts model [17,18,19], which is a generalization of the Ising model [20,21,22]. The authors used the potential of the Potts model in the analysis of distributed connection systems to model the operation of a sensor network.

The Ising model, as well as its development in the form of the Potts model, are perfect for modelling dynamic systems in which the state of individual node may change as a result of the influence of external and internal factors. Examples of such systems are sensor networks, in which changes may occur sporadically, periodically or permanently. Moreover, these models take into account the impact of a given node’s surroundings on its current state, and at the same time a given node itself affects other nodes in its vicinity. The proposed solution uses the properties of both models in the context of distributed sensor systems dedicated i.e., for monitoring. These systems should be characterized by a quick response to critical and alarm events. Examples of such events may be measurements in such places as: forests (fire), rivers (floods), buildings (oxygen level, temperature, humidity), cities (smog), industry (damaged machines, batches of products) and many others. Due to the fact that the Ising model takes into account only two states, the Potts model, taking into account any number of states, constitutes its broader application.

Sensor networks are often based on wireless solutions (the so-called Wireless Sensor Network—WNS), which are battery powered [23,24]. Due to the limited power resources, each process of waking up and then taking measurements significantly affects the life and durability of the sensor. Too frequent activation drains its battery faster. On the other hand, too distant activations in time may cause the omission of important measurements, lack of reading of the alarm state or errors in the subsequent analysis of the results. Therefore, a solution is required to enable effective management of the process of sensor system functioning and their batteries. Obviously, numerous solutions are applied to include these factors in mechanisms dedicated to sensor networks [25,26,27]. The authors decided to use the Potts model for this purpose due to the possibility of taking into account different states. The authors assumed three states for each of the sensors, and also made the value of the Hamiltonian dependent on the exceeding of the limit value of the measured parameter, as well as the state of the battery powering the given sensor node. The adoption of appropriate models enables better understanding of the processes and phenomena occurring in this type of structures, and to study them under various conditions in the future.

It will also enable conducting a series of studies on the susceptibility of individual nodes as well as the entire network to critical situations caused by various events. The use of the presented solution offers the various perspectives of the sensor network—the micro scale, i.e., a single node, as well as the macro scale of the entire infrastructure of interacting elements. Thus, the developed solutions introduce the feature of system adaptability to changing working conditions and develop the analysis of sensor networks towards self-adaptability and self-organization. Considering the sensor network as a complex system, it is also possible to study this type of infrastructure in the future in the context of the system’s operation in the state of equilibrium, on the verge of thermodynamic equilibrium and in the overload state. Additionally, in the future it will be possible to develop the presented approach towards studying various types of anomalies and their impact on the stability of the operation of wireless sensor networks.

Section 2 presents the analysis of the literature in the field of sensor network modelling and the use of the Potts and Ising models to analyze the processes taking place in network structures. Section 3 provides an introduction to the Ising and Potts models. The authors presented their basic properties. Section 4 discusses the proposed method of modelling the interaction between nodes in the sensor network based on the Potts model. Section 5 presents the algorithm developed by the authors for the adopted model. Section 6 provides exemplary simulation results of the developed solution based on an temperature measurement by a network of sensors.

## 2. Analysis of the Literature

The sensor network technology has been developing in many areas. Numerous papers focus on the construction and modelling of such networks, as well as the technologies used. Their modelling is usually based on graph structures. Therefore, methods and means already known from areas such as computer networks, structures of material construction, social and economic relations or biology can be used for this purpose. Recently, tools and methods related to deep learning and artificial intelligence (AI) have also been used. For example, in [28] a PHY-layer authentication scheme was proposed that uses the power delay profile (PDP) of the underwater channels to improve the spoofing detection accuracy. In addition, the authors of this article presented a method based on deep reinforcement learning (DRL). Machine learning algorithms therefore facilitate the analysis and prediction of complex scenarios. Moreover, they enable optimization of functioning not only in the physical layer, but also in the other six layers of the OSI [29]. On the other hand, in [30] the authors formulated a generalized modelling of incorrect data in the problem of localization, which takes into account the influence of both unexpected equipment failures and malicious data falsification. In order to improve localization, the authors proposed a reliable data cleansing algorithm that used low channel bandwidth by source and the rarity of invalid data. In another paper [31], the authors look to the future by presenting a four-tier AI-assisted system architecture to optimize 6G-based network performance. They pointed out that this type of networks with artificial intelligence support will enable intelligent management of mobility, data transmission and spectrum management. On the other hand, the results of the research presented in [32] show the enormous potential of AI in terms of increasing the efficiency of data-focused networks such as Wireless Body Area Networks (WBAN). The use of ML machine learning in them improves accuracy, reliability, scalability, as well as QoS. In the IoT era, the implementation of AI and ML mechanisms is mainly focused on increasing efficiency and capabilities in the application and analytical layers. Another area of research includes solutions related to the effective use of available resources, especially those relating to power. These issues relate to the deployment of IoT devices, including sensors in places where access is limited or even impossible. Therefore, an important aspect of the latest research concerns improving the energy efficiency of this type of equipment. In [33], the authors proposed a distributed power control structure to determine the optimal transmission power for each device. The actions taken involve the requirements of individual devices from the point of view of QoS. In order to achieve the intended goal, coalitions between devices are created, also taking into account the physical limitation of proximity and the quality of the communication channel. Of course, other solutions can be found in the literature regarding the grouping of devices based on the analysis of transmission delays or achievable signal-to-interference plus noise ratio [34,35]. The selected literature items presented above relate mainly to issues increasing the effectiveness of the sensor infrastructure in the analytical and organizational layer, taking into account various parameters, e.g., battery capacity, distance between nodes, etc. However, the solution proposed in this paper refers to modelling of the sensor infrastructure behavior in terms of an effective response to the occurrence of alarm events taking into account internal and external conditions.

However, given the scope of this paper, we will only focus on modelling and curving the Ising and Potts models for this purpose. These models are perfect for describing phase transition occurring in network structures [36,37]. Typically, a phase transition is associated with a rapid and significant change in the values of system parameter. Moreover, these changes are accompanied by a change in the properties of a given system. Analyses of this type mainly relate to the area of physics and electronics, but also to economic and social relations [38,39,40]. However, they are also related to the topological terms of computer networks, including wireless networks [41]. The Ising and Potts models are based on the assumption that the behavior of each node depends on its closest neighbors. They originate from statistical physics, but since they describe the relationships and interactions between nodes accurately, they have been used i.e., for the analysis of social networks [42,43]. However, their potential has also been used in the area of ICT networks. The modification of the Ising algorithm based on the sensitivity factor has been presented in [44]. It focused on the susceptibility to changes of individual nodes in the network. Additionally, the authors used the ordering parameter to visualize phase transition in the network. On the other hand, [45] highlights a completely different area of the model application. The spread of malicious software was analyzed based on the Ising model. The study found that infection becomes faster in less efficient networks with higher data traffic and saturation when the network becomes congested. In [46], the authors presented the possibility of using the Ising model for Medium Access Control (MAC) scheduling in ad hoc networks. The new Hamiltonian proposed by them takes into account the length of the queue and the waiting time on each connection. Consequently, they obtained a low network delay, and increased aggregate throughput and fairness. This paper is an upgrade of previous solutions [47,48]. However, [49] presents the algorithm of adaptive planning of sensor activity based on the Ising model. The authors of this study modelled a distributed sensor network as a random Markov field [50] in a graph that applied the concepts of statistical mechanics to stochastic activation of sensor nodes. The lack of centralized management correlated with local decisions, which in turn led to the global behavior of the sensor network. In turn, the same authors in 2010 [51] presented an interesting approach to adaptive reorganization of the sensor network in order to dynamically adapt to the changing operating environment. This paper, using the Ising model, shows that the sensor network can self-organize, and real-time monitoring and detection is possible thanks to the adaptive redistribution of limited resources. These issues are further developed in [52], where the Ising and Potts models were compared in order to effectively use the wireless sensor network lifetime, as well as to ensure detection and tracking rare and random events. The simulations carried out by the authors showed that the network based on the Potts model has longer life-time than the Ising model, in the same conditions. The obtained results confirmed the great potential of applying the Potts model to the analysis and modelling of the behavior of the WSN network. This solution focuses on individual events and effectively redirects information about them. On the other hand, it does not take into account the level of influence of the node’s surroundings depending on the perception of the current situation (measurements made) by other nodes. A completely different application for the analysis of processes and events in WSN networks was presented in [53]. The authors took into account the important aspect of security and the threat of attacks on the infrastructure of wireless networks. In order to detect potential attacks, they included the monitoring of the behavior of neighboring nodes through the adaptive technique of activating IDS agents (Intrusion Detection systems). They focused on optimizing the activation of these agents on the basis of the Ising model. Another area of application of the Potts model in the modelling and analysis of wireless networks, including sensor networks, is modelling interference in wireless networks [54]. The authors of that paper, based on a real network data, embedded the Potts model in interference plots. The study demonstrated that different interference plots and Potts models clearly influence the degeneration of interference levels.

## 3. Ising and Potts Model Formulation

We are currently witnessing a surge in popularity and development of wireless networks. Due to the growing requirements in the field of reliability, efficiency, throughput, security and scalability, it is necessary to develop new effective solutions both in the field of communication and modelling of their operation. Taking into account the properties of the solution based on the Potts model presented in the paper, such as: scalability, adaptability or responsiveness, the basic communication environment for its application are critical infrastructure networks, Wireless Body Area Networks, MESH networks, and Next-Generation Broadband Wireless Access Networks [55]. These networks have enormous potential to be used in the infrastructure of Smart City, Industry 4.0, autonomous vehicles, telemedicine, textronic systems, etc. All of them may consist of a large number of nodes, each of which performs independent activities, but at the same time is an important element in the entire infrastructure. Moreover, decisions made locally may also be of regional importance (they affect the surroundings of a given node), and even in the context of the entire network. Additionally, the indicated networks in many cases will make independent measurements of various parameters, and their value will determine further actions. Therefore, we decided to use the Potts model in our work.

As already mentioned, the Ising and the Potts model are models derived from the theory of statistical physics, but at the same time they relate to the processes taking place in the structures of complex systems. These processes can result from both internal and external influences. The purpose of these models is to study the reaction of the system, and in particular its individual elements in terms of interaction. The accompanying processes can be both micro and macroscopic due to the fact that these interactions result from the current state characteristic of individual elements, but also from the states characteristic of their environment. Therefore, modelling based on these models is associated with both the entire system and its individual elements. In these models, a given system is mapped in the form of a network structure, usually described as a graph, where nodes denote system elements, and a set of edges denote relations between them.

It should be noted that the basic model is the Ising model. This classic model is based on the assumption that each node (spin) can take one of the two values +1 or −1, i.e., $s_{i}\in \left\{-1,1\right\}$. In that case, the energy of any state is given through the Hamiltonian of the system [20,21]:

(1) $$H=-\sum_{\left\langle i,j\right\rangle }J_{ij}s_{i}s_{j}-B\sum_{i}s_{i},$$

where *s_i_* stands for the state of *i*-th node, *J* is the coupling constant between a node *v_i_* and *v_j_*, while *B* is an external magnetic field. In most cases of modelled network structures, including IT ones, the external field described by *B* is neglected. Additionally, considering that all neighbors of *v_i_* have the same impact force, we can adopt another simplification:

(2) $$H=-J\sum_{\left\langle i,j\right\rangle }s_{i}s_{j}.$$

Note that the Ising model was originally about models of magnetism. Therefore, adequate values were adopted for both states. However, different values of the states are introduced that each of the nodes can assume in implementations of the Ising model in other areas. However, this model is also of limited use due to the limitation to 2 states. Therefore, the Potts model is its extension. In this model, it is possible to define a wider set of available states. Let us denote this set as $S=\left\{s_{1},s_{2},\ldots ,s_{q}\right\}$ and it is a finite set, where q∈ℤ. Then, on the basis of (2) we obtain:

(3) $$H=-J\sum_{\left\langle i,j\right\rangle }\delta \left(s_{i}s_{j}\right),$$

where $\delta \left(s_{i}s_{j}\right)$ is Kronecker delta which equals one whenever *s_i_* = *s_j_* and zero otherwise. When considering two separate states *s*_1_ and *s*_2_, as well as the probability of their occurrence as $p\left(s_{1}\right)$ and $p\left(s_{2}\right)$-the relative probability that the system is in two states is:

(4) $$p=\frac{\;p\left(s_{2}\right)}{p\left(s_{1}\right)}=\frac{e^{\beta H_{2}}}{e^{\beta H_{1}}},$$

where β=1/kT parameter specified by the absolute temperature *T* and the Boltzmann constant *K*, and *H*_1_ is Hamiltonian of state *s*_1_, and *H*_2_ is Hamiltonian of state *s*_2_. Due to the fact that the considered system is independent of temperature, we assume that if p≥1 then the node changes state from *s*_1_ to *s*_2_, otherwise, the node remains in state *s*_1_. Therefore, the probability of the occurrence of a node state change can be briefly written as $p_{flip}=max\left(1,p\right)$.

## 4. Suggested Method

Let us assume, there is a graph G={V,E,W}, where *V* denotes a set of vertices (sensors), *E* set of edges, and *W* set of edge weights. Every $w_{ij}\in W$ denotes the relation between the vertex *v_i_* and the vertex *v_j_*. Then the Expression (3) takes the form:

(5) $$H=-w\sum_{\left\langle i,j\right\rangle }\delta \left(s_{i}s_{j}\right).$$

It should be remembered that the graph *G* can be a directed graph if $\left(v_{i},v_{j}\right)\neq \left(v_{j},v_{i}\right)$, and what is more, the relation between neighbors can be variable in space and time. In this case, based on the Expression (2) and taking into account (5) we get:

(6) $$H=-\sum_{\left\langle i,j\right\rangle }w_{ij}\left(\tau \right)\delta \left(s_{i}s_{j}\right),$$

where $w_{ij}\left(\tau \right)$ is the value of the relation between the vertex *v_i_* and the vertex *v_j_* at time *τ*. In the case of the Ising model, we can assume the following states for individual sensors:

(7) $$s_{i}=\{\begin{array}{cc} s_{1} & \mathrm{when}\;\mathrm{the}\;\mathrm{node}\;\mathrm{is}\;\mathrm{in}\;\mathrm{an}\;\mathrm{active}\;\mathrm{state}\\ s_{2} & \mathrm{when}\;\mathrm{the}\;\mathrm{node}\;\mathrm{is}\;\mathrm{in}\;\mathrm{an}\;\mathrm{inactive}\;\mathrm{state} \end{array}.$$

However, taking into account the Potts model, we introduce the following 3 states:

(8) $$s_{i}=\{\begin{array}{cc} s_{1} & \mathrm{when}\;\mathrm{the}\;\mathrm{node}\;\mathrm{is}\;\mathrm{in}\;\mathrm{an}\;\mathrm{active}\;\mathrm{state}\;\\ s_{2} & \mathrm{when}\;\mathrm{the}\;\mathrm{node}\;\mathrm{is}\;\mathrm{in}\;a\;\mathrm{standby}\;\mathrm{state}\\ s_{3} & \mathrm{when}\;\mathrm{the}\;\mathrm{node}\;\mathrm{is}\;\mathrm{in}\;\mathrm{an}\;\mathrm{inactive}\;\mathrm{state}\; \end{array}.$$

Then dependencies should be determined $\delta \left(s_{i}s_{j}\right)$:

(9) $$\delta \left(s_{i}s_{j}\right)=\{\begin{array}{cc} 1 & s_{i}=s_{j}\\ 0,5 & s_{i}\neq s_{j},\;\;s_{j}\;\mathrm{is}\;\mathrm{in}\;a\;\mathrm{standby}\;\mathrm{or}\;\mathrm{active}\;\mathrm{state}\\ 0 & s_{i}\neq s_{j},\;\;s_{j}\;\mathrm{is}\;\mathrm{in}\;\mathrm{an}\;\mathrm{inactive}\;\mathrm{state}\; \end{array}.$$

**Figure 1:**
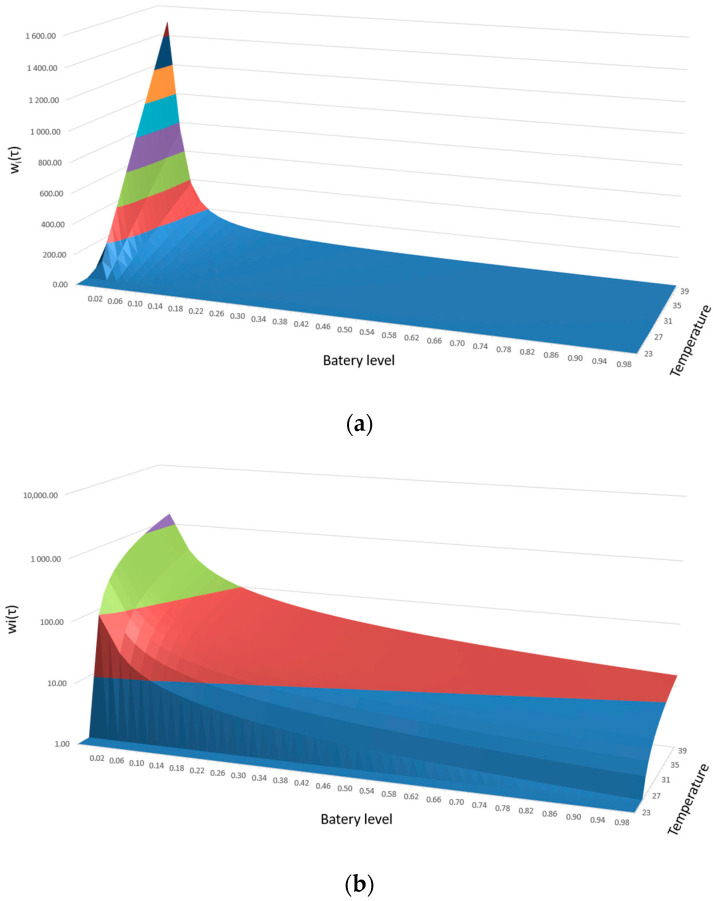
The dependence of the impact of *w_i_(τ)* of a given node on the temperature increase and battery level; (**a**) standard chart; (**b**) logarithmic chart.

In this paper it was assumed that each sensor (node) carries out one type of measurement. This measurement refers to the variable m(τ), which can assume at different times τ∈T different values from the set $\;M=\left\{m_{i}\left(\tau \right)\in \mathbb{R}\right\}$, where $m_{i}\left(\tau \right)$ is the value of the parameter *m* measured in time *τ* by the node *v_i_*. Nodes have defined threshold values for their $m_{i}^{k}\in M\;$ measurements. Exceeding of the threshold value shall be linked to the value of the interaction between nodes. For this purpose, the value of the impact of a given node *v_i_* was introduced using the expression:

(10) $$w_{i}\left(\tau \right)=\frac{\left|m_{i}^{k}-m_{i}\left(\tau \right)\right|}{b_{i}\left(\tau \right)},$$

where $b_{i}\left(\tau \right)\subset \left|0\;;\;1\right\rangle$ is the battery level of node *v_i_*. The greater the exceeding of the threshold value and the more discharged the battery, the faster the impact on the neighboring node. To better illustrate this relation, Figure 1 presents an exemplary influence of the value of the impact of a given node on the increase of the measured temperature above the adopted threshold value (in this case 25 °C) and of course on the value of the battery level. Due to the fact that the standard representation of the obtained values (Figure 1a) does not illustrate the dynamics of the increase in the value of the impact of a given node, Figure 1b presents the obtained results in a logarithmic scale.

In order to take into account the influence of exceeding the threshold parameters on the force of interaction between nodes expressed by *w_ij_*, and then on the Hamiltonian (6), the following relationship was introduced:

(11) $$w_{ij}=\{\begin{array}{cc} 1 & \mathrm{if}\;m_{l}\left(\tau \right)<m_{l}^{k}\;\left(l=i,j\right)\;\\ w_{i}\left(\tau \right) & \mathrm{if}\;\mathrm{for}\;v_{i}\;m_{i}\left(\tau \right)\geq m_{i}^{k}\\ -w_{j}\left(\tau \right) & \mathrm{if}\;\mathrm{for}\;v_{i}\;m_{i}\left(\tau \right)<m_{i}^{k},\;\mathrm{and}\;m_{j}\left(\tau \right)\geq m_{j}^{k} \end{array}.$$

Expression (11) allows to take into account various situations taking place in the sensor network. The first case concerns the situation when *w_ij_* takes the value 1. It is a neutral value from the point of view of the calculated value of Hamiltonian and applies to the situation when the value of the measurements for both nodes is within the norm and both nodes remain inactive. However, if during the measurement for a given node *v*_i_ it reads a value that exceeds the threshold state for it (then it is in the active or standby state), it is reinforced by the value *w_i_*(*τ*) compared to any other states in neighboring nodes that are not to enumerate this expression. On the other hand, if the node neighboring *v_j_* reads a value exceeding its threshold state, then the interaction of the node *v_j_* should be strong enough to weaken the state of the inactive node *v_i_*. This can be compared to the neighbor node excitation signal and is expressed as a negative value *w_j_*. As a result, in the Potts model, node *v_i_* will have less “willingness” to remain in its current state. The threshold value itself can have different values and be adjusted to the actual measurement needs. The adopted model does not limit what the value is to be and what specific measurements it should refer to. Therefore, this model is universal and can be used in various areas. Of course, the difference between the actual read value and the set threshold value is significant. Thus, a greater exceeding of the threshold has a faster and greater impact on a neighboring node according to Expressions (10) and (11). Additionally, the force of the impact is enhanced by the battery charge level. In the proposed model, the battery capacity does not influence the alarming efficiency. Alarm events result from exceeding the set threshold value. Of course, the capacity of the battery may indirectly influence the strength of the internode interaction by relating to the current level of the battery. However, in this solution it is assumed that all nodes have the same battery capacity. The basis for this assumption was the fact that most of this type of sensor structures is homogeneous, e.g., in terms of the battery capacities used. In the future, separate studies on the impact of different battery capacities on the functioning of the adopted model may be conducted taking into account different battery charging systems (local and remote).

## 5. Developed Algorithm

The method proposed in Section 4 was used in the development of the sensor network algorithm based on the assumptions of the Potts model. For this purpose, it was assumed that a given structure of a wireless sensor network consists of *N* nodes. These nodes are distributed over an area of *L* × *L*. Within these areas, such cliques *C_i_* are created so that the clique is formed by nodes that “see” each other, i.e., are within the range of their radio signal. Only the nodes in a given clique interact with each other and are considered in each iteration. Obviously, a given node may belong to several cliques and thus adopt states from different cliques, but its state is analyzed only from the perspective of its clique, i.e., nodes that are within its range. Within the clique, the nodes are in active, inactive or standby state. The devices take measurements in all possible states. It was assumed that individual states characterize the further operation and behavior of each device as follows:

- Inactive state—the device performs a measurement at every set interval of time (e.g., every 15 s). When the set limit value is exceeded or equalized, it changes its state to standby.
- Standby state—the device performs a measurement at a set period every one time unit (e.g., 5 measurements within 5 s). If, any successive value falls below the set alarm threshold $m^{k}$ during this measurement period, the device returns to the inactive state. When, after a predetermined period, all the measurements are equal to or above the threshold value, the device turns into the active state.
- Active state—the device performs continuous measurements every one time unit as long as the measured value equals or exceeds the alarm value $\;m^{k}$. When the measured value is below the set threshold value, this device will change its state to standby.

**Figure 2:**
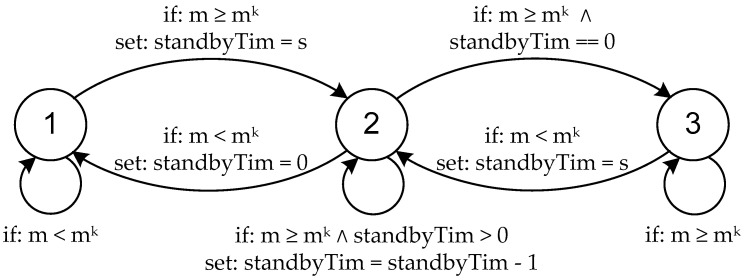
State machine presenting the transitions between individual states of nodes.

Figure 2 shows a state machine reflecting the transitions between the individual states of nodes. For the assumptions made above, let *m* is the measurement taken, *m^k^* is the limit value, standbyTim is the standby time counter, *s* is the time the node remains in the standby state. Whereas 1 is inactive, 2 is in standby and 3 is active. Of course, the adopted model is universal and flexible, i.e., it allows to adopt different limit parameters and their values, as well as the intensity of measurements. The above-mentioned assumptions made by the authors made it possible to carry out specific tests and simulations.

Based on the adopted model, an algorithm for simulating the operation of the sensor network was developed, the diagram of which is presented in Listening 1.
**Listening 1.** Sensor network simulation algorithm.**Input:** An attributes-value *N*, *L*. 1.  Enter parameters: simulation time *time*, current simulation step t=0.2.   Creating a sensor network G={V,E}.3.  Generating a map of the measured environment.4.  **For each** node $v_{i}\in V$:5.    For each node, check whether it is in the active state, standby or there is another measurement period of inactive state.6.    **If** NO **Then** set inactive timer in the device before the next measurement7.       **Else** perform the following steps:8.       - Get $m_{i}\;$ values from the latest generated map of the environment being measured.9.       - Update the node state based on $m_{i}\;$ measurement and state machine.10.       - Calculate the Hamiltonian *H*.11.       - Decrement the node’s battery level.12.  **End For**13.  For each pair of nodes $\left(v_{i},v_{j}\right)\;$ in cliques, call the function $\;flip\left(s_{i},s_{j}\right)$.14.  Decrement all node counters: standby timer, inactive timer.15.  Save the results of the current simulation step.16.  **If**
t<time
**THEN** come back to step 2 17.    **Else** plot a graph and simulation results.18.  Complete the sensor network simulation algorithm.

The creation of a sensor network is based on the number of nodes *N* and the size of the area *L* × *L*. However, the presented algorithm does not limit the way of creating individual structures and the way of arranging individual nodes. For example, the methods of random, even distribution can be used based on the theory of complex systems (small worlds, power law) etc., but the predetermined exact distribution of nodes can be also taken into account. Generating a map of the measured environment is related to the adoption of constant or variable data that are subject to measurement. It can be a map of the distribution of pressure, temperature, gas concentration, etc. The assumption is that the size of this map should correspond to the sensor location. Section 6 presents i.e., an exemplary heat map adopted as a part of the simulations. Function *flip*(*s_i_,s_j_*) allows to change the state of a given node based on the current value of the Hamiltonian and the Expression (4).

To offer more thorough analysis of the proposed solution, its computational complexity was also determined. Thus, it has a complexity of O(*3n*) for each step, where *n* is the number of nodes. This is due to from the fact that no nodes were favored in the order of computation. In the analyzed aspect, three basic stages can be distinguished: measurement and setting of states, calculation of the Hamiltonian, state change. Due to the fact that the constants should be omitted in this case, the computational complexity is then O(*n*). Additionally, computation time analyzes were performed for a specific number of nodes and simulation steps (Table 1). Of course, these are only illustrative values, because they were implemented for specific network structures and a common heat map, so that the results could be compared. The hardware platform on which the simulations are performed also affects the results. In this case it was an Intel (R) Core (TM) i5-42150H CPU @ 2.9GHz (4 CPUs) computer and 8192 MB of RAM. It should also be remembered that the simulation environment includes calculations for all nodes, and in the real environment the calculations are distributed among individual nodes. Nevertheless, it can be observed that the simulation time increases as the number of simulation steps increases. Additionally, an important element determining the simulation time is the moment of exceeding the threshold value, because then the calculations related to i.e., with a change in the current state of individual nodes.

## 6. Simulations

The proposed solution based on the Potts model has been implemented using the Python language. This language was chosen due to its wide functionality, flexibility and efficiency in the field of data processing, as well as to ensure the possibility of future development of a given software towards the use of artificial intelligence and machine learning mechanisms. Of course, the presented solution and the algorithm itself can be implemented in any software environment.

**Figure 3:**
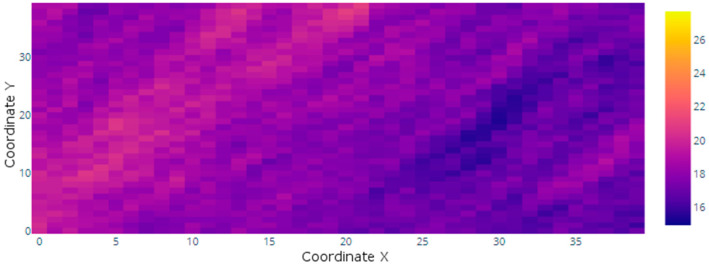
Sample heat map.

One of the basic elements that influence the course of the simulation is defining a map of the measured environment. As previously emphasized, maps may refer to various measured parameters. To demonstrate the functioning of the developed model, the authors used a heat map representing the temperature distribution. An example of such a map is shown in Figure 3. The lower the temperature, the darker the color.

In their considerations, the authors assumed three different simulation approaches to generating data constituting the basis of the heat map:

- Heat map with a random gradient—the algorithm changes the values on the heat map completely randomly as a function of time.
- Heat map with cyclical changes in value—this algorithm increases or lowers the values of the heat map by a set coefficient in cyclic periods.
- Heat map with zones of temperature changes—this algorithm changes a certain circular area of the heat map by a given coefficient for a certain simulation period.

Of course, the three simulation variants proposed by the authors are not the only possible ones. The available approaches also include the registration of the actual measurement values in a given area, as well as the implementation of other models. The presented results of the experiment refer to the environment consisting of 50 independent nodes located in the environment with dimensions of 40/40 units. One unit can correspond to a distance of 1 m. For the heat map, the data was generated using the algorithm “Heat map with a random gradient”. In this specific case, the temperature threshold was set at 20 °C. The simulation was run with a limitation of 1000 steps, and each simulation step corresponded to 1 s. The standby time was set to five simulation steps. Nodes in the inactive state performed the measurement every 10 s. These values are specific for a given simulation, while the adopted model and algorithm, as well as the simulation environment enable their free selection to the current simulation needs.

**Figure 4:**
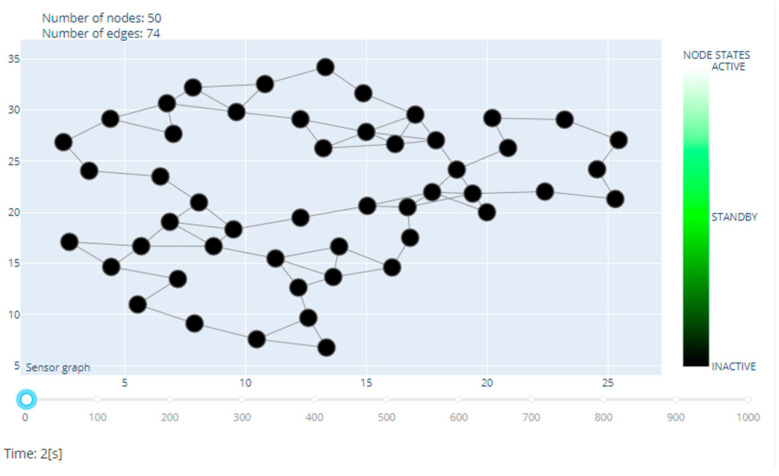
An example of the structure of a simulated sensor network.

An exemplary structure of a sensor network consisting of 50 nodes is shown in Figure 4.

**Figure 5:**
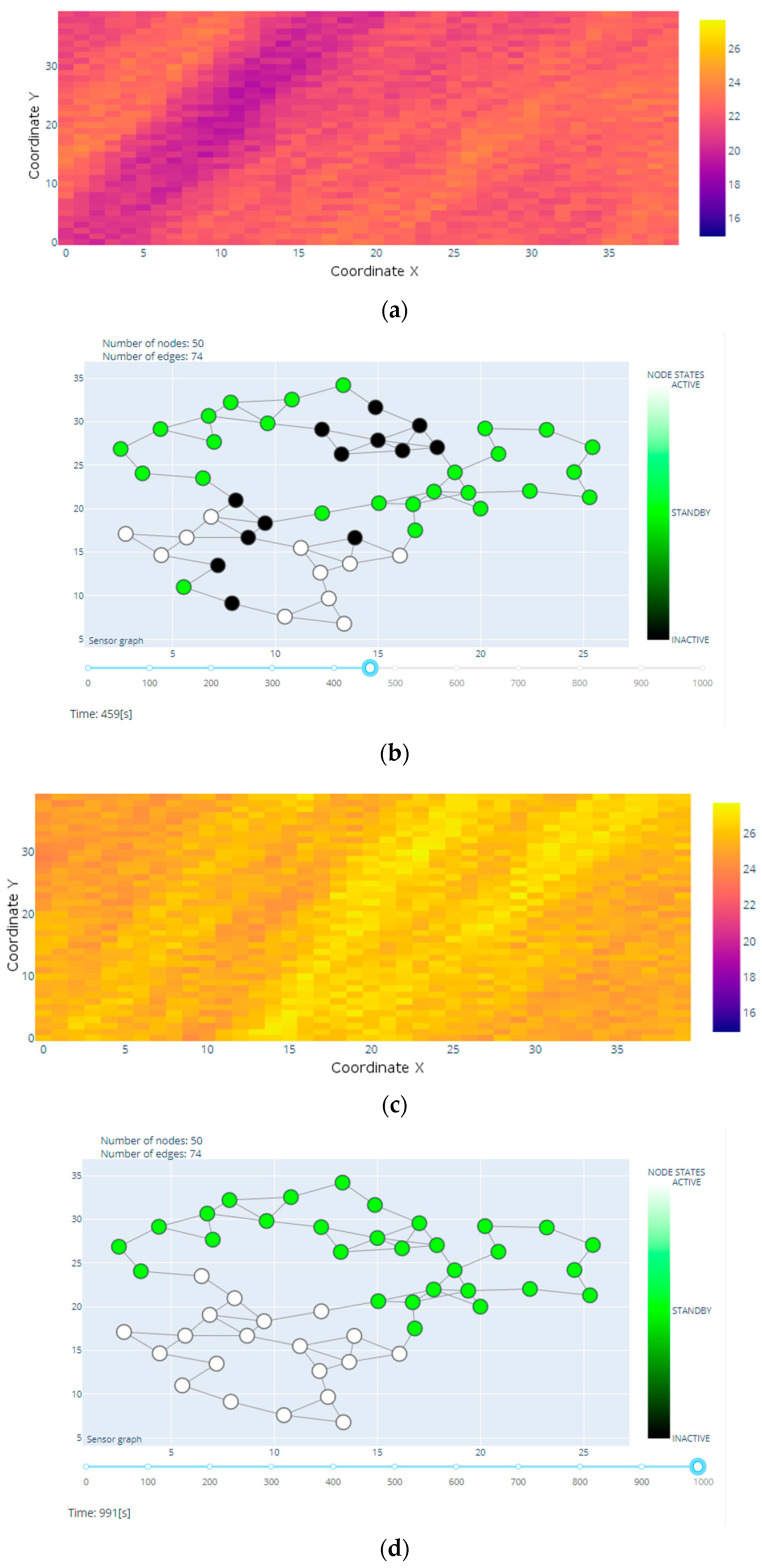
Selected steps of the simulation; (**a**) heat map in 459 s; (**b**) states of individual nodes in 459 s; (**c**) heat map in 991 s; (**d**) states of individual nodes in 991 s.

In the presented example, in the initial phase of the simulation, all nodes were in an inactive state based on the current temperature measurements. Along with the simulated temperature changes in a given area causing the accepted threshold value to be exceeded, the states of individual nodes also change. These changes depend on the current value of the Hamiltonian and the developed model. Two selected simulation steps illustrating these changes are presented in Figure 5.

**Figure 6:**
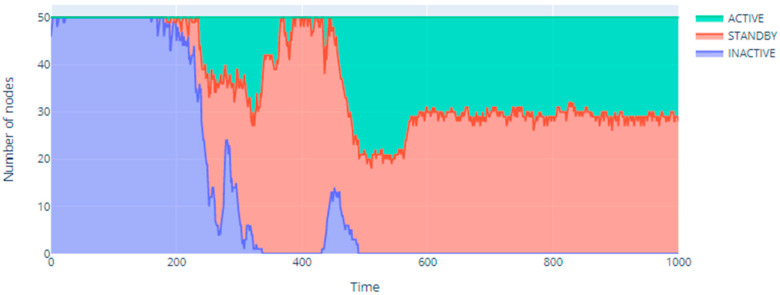
Distribution of node states throughout the simulation.

Interesting results are shown in Figure 6, which presents the number of nodes in a given state in successive steps of the simulation. It clearly shows the phase transition taking place in the infrastructure of the sensor network [41]. This is one of the characteristics of complex systems. It is associated with a rapid change in the values of important parameters of a given object or system, in this case the network infrastructure. As a result of phase transitions, a given network also changes its properties. These changes can be caused by both internal and external factors. In the case under consideration, they refer to significant changes in the ambient temperature that directly affect the current state of individual nodes. Thus, the phase transitions perfectly reflect significant structural changes in network systems, including the relations between individual nodes of these structures. According to the assumptions, the Potts model, like the Ising model, perfectly reflects the processes of phase transitions. Thus, in Figure 6 it can be seen that the entire network is initially stable, then there is a phase transition during which the network is unstable and then the network structure stabilizes again. During the phase transition period, a changing distribution of all available states is visible, in which each of the nodes can be. However, in the end, two states remain in the example—active and standby. The phase transition reflects the dynamics of the system (network). Typically, one of the basic effects of the phase transition is the stabilization of the system and the limitation of the active states of individual nodes, which is also observed in Figure 6. In the example presented, the phase transition period takes place in the range of approx. 160–570 s simulations.

**Figure 7:**
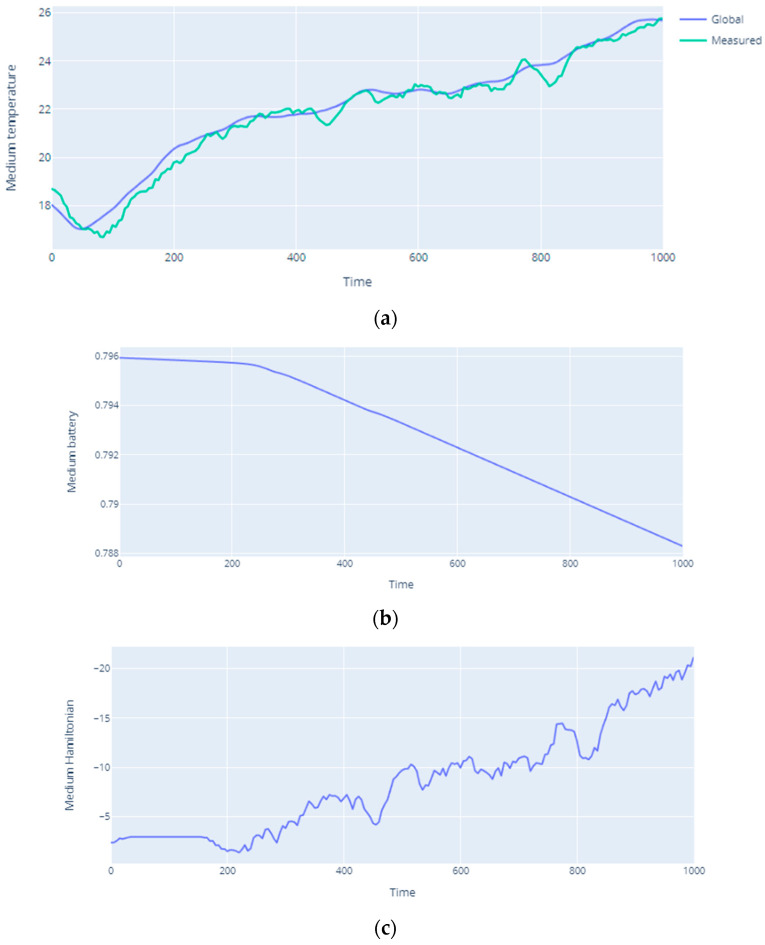
(**a**) Change in average temperature over time for the entire area and at measurement points; (**b**) battery level during simulation; (**c**) change of Hamiltonian value during simulation.

The compliance of the assumptions of the presented model with the obtained results can be seen in Figure 7. Exactly, in Figure 7c, the value of the Hamiltonian increases during the simulation. It is related to the influence of exceeding the threshold parameters on the strength of interaction between nodes expressed by *w_ij_,* and thus on the Hamiltonian. This value is directly influenced by the temperature and the battery level according to Expressions (10) and (11). Therefore, the temperature increase (Figure 7a) and the decrease of the battery level (Figure 7b) according to Expressions (10) and (11) determine the value of the Hamiltonian (11). Thus, according to the assumptions adopted in Section 4, the growth rate of the difference between the threshold value and the measured value is directly related to the dynamics of phase changes occurring in the system. Comparing Figure 7a with Figure 6, it can be seen that when the assumed threshold value is exceeded (in the considered case 20 °C), around 190s of the simulation, the Hamiltonian value increases and the phase transformation process begins. Figure 7a shows that in the considered case the global temperature change over the entire area is very close to the values of measurements made by individual nodes at specific points of their location. Effective battery management, especially in the inactive state, can be seen in Figure 7b, which shows the average battery level in the entire infrastructure at individual simulation steps. At the beginning of the simulation, most nodes were inactive, so the battery drains much slower than from about 250–300 s, where the battery discharge rate is much faster. It was then that more nodes began to take measurements more actively. This is partially visible in Figure 7c as the battery level also affects the Hamiltonian value.

**Figure 8:**
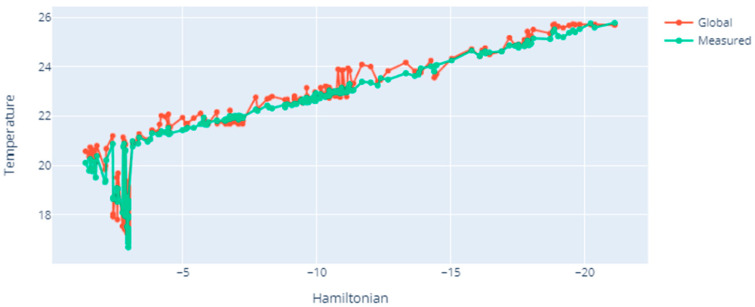
Dependence of Hamiltonian value on temperature.

Figure 8 presents exemplary simulation results in terms of the Hamiltonian dependence on temperature. In the case under consideration, the increase in temperature directly influences the increase of the Hamiltonian value. Thus, as the temperature rises, the Hamiltonian value increases. As in Figure 7a, in this case the global temperature change over the entire area follows a similar course as point measurements made by individual nodes at specific points of their location. Thus, the proper placement of the sensors, and then their measurements in a given area, reflected the global temperature distribution very well in the considered cases.

The solution proposed in the study is characterized by high scalability, which results from the assumptions underlying the model itself and the developed algorithm. Due to the fact that decisions regarding a possible change of the state of a given node depend only on its current state, local parameters and states of nodes located in its immediate vicinity, and thus the Hamiltonian calculations are also performed locally, theoretically, there is no limitation in terms of size the structure under consideration. From the point of view of a single node, the proposed solution does not hold the entire topology or routing table. Of course, from the point of view of the simulation environment, the limitation may constitute table sizes resulting from the number of nodes and analyzed parameters that can be stored and processed by a given simulation environment. In the context of the scalability of the proposed solution in the simulation environment, one can take into account the computational complexity of the developed algorithm, the analysis of which is presented in Section 5. However, in the real environment, the implementation of the presented model would also not face any scalability limitations, as possible limitations would rather result from applied technological solutions, e.g., the number of possible connections between a given node and its neighbors. Thus, a significant increase in the number of nodes in the actual network would not determine the necessity to use a significantly larger RAM or additional processors, since the computations were performed in a distributed environment.

## 7. Conclusions

Sensor networks constitute and will constitute a very important area of functioning of smart homes and smart buildings, smart cities, smart factories, military, warning systems, autonomous vehicles, etc. The development of this field of science and technology is very fast, both in terms of technical solutions and in the area of systems and applications supporting their functioning. An important aspect is also the methods and means of modelling the behavior of this type of network. Thanks to them it is possible to simulate and test their operation, which allows to study the processes taking place in them, and then to improve and develop them in order to increase their efficiency. The solution developed by the authors based on the Potts model takes into account 3 states in which each of the nodes can be located. The change in the state of individual nodes depends on their current state and the values of the measured parameters, as well as on the influence of neighboring nodes on them. For this purpose, the authors proposed a solution that takes into account the dependence of the Hamiltonian value on the exceeding of the limit value of the measured parameter (corresponding to an alarm event), but also on the state of the battery supplying a given sensor node. The model adopted in this way increases the efficiency of the sensor network from the point of view of saving power sources. This aspect is particularly important in the context of this type of network. The advantage of the presented solution is taking into account the level of influence of the surroundings of a given node in the context of the current situation perceived by this environment (based on measurements made by other nodes). In this way, the effectiveness of the measurements is increased, which can directly affect their precision.

The authors pointed out that sensor networks should be considered from the point of view of complex systems. One of the characteristic properties of these systems are the phase transitions that occur in them. The assumptions adopted by the authors of the work and the model developed by them allow for taking into account this type of phenomena and then their observation. A separate issue is the future research on the impact of such phenomena on the stability of the sensor network infrastructure.

An additional area of further research concerns the inclusion of external influence in the proposed model. Thanks to this, it would also be possible to study the influence of macroscopic factors on the functioning of sensor networks. The authors believe that this research direction may bring us closer to obtaining a model that corresponds to a wider extent to real complex systems.

## Figures and Tables

**Table 1:** The real computation time [s] for a given number of nodes and simulation steps.

Number of Nodes	Number of Steps			
	100	1000	5000	10,000
20	<1	1	23	51
40	<1	3	65	93
60	1	6	98	131
80	1	6	132	193

## References

[1] Lu Y. (2017). Industry 4.0: A survey on technologies, applications and open research issues. J. Ind. Inf. Integr..

[2] Schütze A., Helwig N., Schneider T. (2018). Sensors 4.0—Smart sensors and measurement technology enable Industry 4.0. J. Sens. Sens. Syst..

[3] Silva M., Vieira E., Signoretti G., Silva I., Silva D., Ferrari P. (2018). A Customer Feedback Platform for Vehicle Manufacturing Compliant with Industry 4.0 Vision. Sensors.

[4] Duarte M., Hu Y.H. (2004). Vehicle classification in distributed sensor networks. J. Parallel Distrib. Comput..

[5] Pietrelli A., Micangeli A., Ferrara V., Raffi A. (2014). Wireless Sensor Network Powered by a Terrestrial Microbial Fuel Cell as a Sustainable Land Monitoring Energy System. Sustainability.

[6] Ibrahim M., Moselhi O. (2014). Wireless Sensor Networks Configurations for Applications in Construction. Procedia Eng..

[7] Hughes-Riley T., Dias T. (2018). Developing an Acoustic Sensing Yarn for Health Surveillance in a Military Setting. Sensors.

[8] Boubiche D.E., Pathan A.-S.K., Lloret J., Zhou H., Hong S., Amin S.O., Feki M.A. (2018). Advanced Industrial Wireless Sensor Networks and Intelligent IoT. IEEE Commun. Mag..

[9] Furtak J., Zieliński Z., Chudzikiewicz J. Security techniques for the WSN link layer within military IoT. Proceedings of the IEEE 3rd World Forum on Internet of Things (WF-IoT).

[10] Ramson S.R.J., Moni D.J. Applications of wireless sensor networks—A survey. Proceedings of the International Conference on Innovations in Electrical, Electronics, Instrumentation and Media Technology (ICEEIMT).

[11] Auyang S.Y. (1999). Foundation of Complex-System Theories.

[12] Boccara N. (2010). Modeling Complex Systems.

[13] Ladyman J., Lambert J., Wiesner K. (2013). What is a complex system?. Eur. J. Philos. of Sci..

[14] Grabowski F. (2013). Nonextensive model of self-organizing systems. Complexity.

[15] Comin C.H., Peron T., Silva F.N., Amancio D.R., Rodrigues F.A., Costa L.D. (2020). Complex systems: Features, similarity and connectivity. Phys. Rep..

[16] Weisbuch G. (2018). Complex Systems Dynamics.

[17] Wu F.Y. (1982). The Potts model. Rev. Mod. Phys..

[18] Ashkin J., Teller E. (1943). Statistics of Two-Dimensional Lattices with Four Components. Phys. Rev..

[19] Dorogovtsev S., Goltsev A.V., Mendes J.F. (2004). Potts model on complex networks. Phys. Condens. Matter.

[20] Fisher D.S., Fröhlich J., Spencer T. (1984). The Ising model in a random magnetic field. J. Stat. Phys..

[21] Cipra B.A. (1987). An Introduction to the Ising Model. Am. Math. Mon..

[22] Zhang Z. (2020). Computational complexity of spin-glass three-dimensional (3D) Ising model. J. Mater. Sci. Technol..

[23] Antolín D., Medrano N., Calvo B., Pérez F. (2017). A Wearable Wireless Sensor Network for Indoor Smart Environment Monitoring in Safety Applications. Sensors.

[24] Mekonnen T., Porambage P., Harjula E., Ylianttila M. (2017). Energy Consumption Analysis of High Quality Multi-Tier Wireless Multimedia Sensor Network. IEEE Access.

[25] Alippi C., Galperti C. (2008). An Adaptive System for Optimal Solar Energy Harvesting in Wireless Sensor Network Nodes. IEEE Trans. Circuits Syst. I Regul. Pap..

[26] Silva A., Liu M., Moghaddam M. (2012). Power-Management Techniques for Wireless Sensor Networks and Similar Low-Power Communication Devices Based on Nonrechargeable Batteries. J. Comput. Netw. Commun..

[27] La Rosa R., Livreri P., Trigona C., Di Donato L., Sorbello G. (2019). Strategies and Techniques for Powering Wireless Sensor Nodes through Energy Harvesting and Wireless Power Transfer. Sensors.

[28] Xiao L., Sheng G., Wan X., Su W., Cheng P. (2019). Learning-Based PHY-Layer Authentication for Underwater Sensor Networks. IEEE Commun. Lett..

[29] Huang X.L., Ma X., Hu F. (2018). Machine Learning and Intelligent Communications. Mob. Netw. Appl..

[30] Kan C., Ding G., Wu Q., Zhang T. (2018). Robust Localization with Crowd Sensors: A Data Cleansing Approach. Mob. Netw. Appl..

[31] Yang H., Alphones A., Xiong Z., Niyato D., Zhao J., Wu K. (2020). Artificial Intelligence-Enabled Intelligent 6G Networks. IEEE Netw..

[32] Al-Turjman F., Baali I. (2019). Machine learning for wearable IoT-based applications: A survey. Trans. Emerg. Telecommun. Technol..

[33] Tsiropoulou E.E., Paruchuri S.T., Baras J.S. Interest, Energy and Physical-Aware Coalition Formation and Resource Allocation in Smart IoT Applications. Proceedings of the 51st Annual Conference on Information Sciences and Systems (CISS).

[34] Tefek U., Lim T.J. Clustering and radio resource partitioning for machine-type communications in cellular networks. Proceedings of the IEEE Wireless Communications and Networking Conference.

[35] Luan X., Wu J., Wang B., Cheng Y., Xiang H. Distributed network topology formation and resource allocation for clustered Machine-to-Machine communication networks. Proceedings of the 11th International Conference on Wireless Communications, Networking and Mobile Computing (WiCOM).

[36] Stanley H.E. (1989). Introduction to Phase Transitions and Critical Phenomena.

[37] Perc M. (2016). Phase transitions in models of human cooperation. Phys. Lett. A.

[38] Wang B., Han Y., Chen L., Aihara K. (2009). Multiple Phase Transitions in the Culture Dissemination. Complex Sci..

[39] Hors I., Lordon F. (1997). About some formalisms of interaction Phase transition models in economics?. J. Evol. Econ..

[40] Onuki A. (2002). Phase Transition Dynamics.

[41] Paszkiewicz A., Bolanowski M., Zapała P. (2019). Phase Transitions in Wireless MESH Networks and Their Application in Early Detection of Network Coherence Loss. Appl. Sci..

[42] Liu S., Ying L., Shakkottai S. Influence maximization in social networks: An ising-model-based approach. Proceedings of the 48th Annual Allerton Conference on Communication, Control, and Computing.

[43] Suchecki K., Hołyst J.A. (2006). Ising model on two connected Barabasi-Albert networks. Phys. Rev. E.

[44] Paszkiewicz A., Iwaniec K. Use of Ising Model for Analysis of Changes in the Structure of the IT Network. Proceedings of the 39th International Conference on Information Systems Architecture and Technology (ISAT).

[45] Antonio K.E.S., Pinol C.M.N., Banzon R.S. An Ising Model Approach to Malware Epidemiology. https://arxiv.org/abs/1007.4938.

[46] Rahman T.A.U., Hassan M.S., Ismail M.H. (2020). A Novel Medium Access Control Algorithm for Ad Hoc Networks Based on Ising Model. IEEE Access.

[47] Wang Y., Xia Y. (2018). I-CSMA: A link-scheduling algorithm for wireless networks based on Ising model. IEEE Trans. Control Netw. Syst..

[48] Li X., Tolmachev P., Pauley M., Manton J.H. A distributed transmission scheduling algorithm for wireless networks based on the ising model. Proceedings of the IEEE Statistical Signal Processing Workshop (SSP).

[49] Srivastav A., Ray A., Phoha S. (2009). Adaptive Sensor Activity Scheduling in Distributed Sensor Networks: A Statistical Mechanics Approach. Int. J. Distrib. Sens. Netw..

[50] Perreau S., Sigelle M., Da Silva P., Jayasuriya A. Sensor Networks Protocol Design Using Random Markov Field Theory. Proceedings of the 6th Annual IEEE Communications Society Conference on Sensor, Mesh and Ad Hoc Communications and Networks.

[51] Srivastav A., Ray A. (2010). Self-organization of sensor networks for detection of pervasive faults. Signal Image Video Process..

[52] Assa A., Jahan M.V. Adaptive scheduling in wireless sensor networks based on Potts model. Proceedings of the 2nd International eConference on Computer and Knowledge Engineering (ICCKE).

[53] Hai T.H., Huh E.N. Optimal Selection and Activation of Intrusion Detection Agents for Wireless Sensor Networks. Proceedings of the Future Generation Communication and Networking (FGCN 2007).

[54] McBride N., Bulava J., Galiotto C., Marchetti N., Macaluso I., Doyle L. (2017). Degeneracy estimation in interference models on wireless networks. Phys. A Stat. Mech. Appl..

[55] Singhal C., De S. (2017). Resource Allocation in Next-Generation Broadband Wireless Access Networks.

